# Infective Inflammation of the Parotid Glands

**Published:** 1896-04

**Authors:** 


### Infective Inflammation of the Parotid Glands.

The *Journal de Med. de Paris* of January 19th, 1896, contains a study of three cases of this kind, interesting on account of their etiology and treatment, by Regnier, surgeon at the Lariboisere. He says that until recently we have always considered these parotid inflammations as caused by some secondary inflammation of the lymphatic glands. We are now becoming aware of the fact that it is the glandular pockets of the gland itself that are involved, resulting from a primary inflammation of the excretory canal. The infection starts in the mouth and passes up through the excretory canal to the pockets of the gland.

Chassaignac first called attention to this variety, which he called canalicular, characterized by swelling of the parotid region, followed by pus discharging through the duct of Steno. Virchow and Weber also observed cases where the pus and the lesions were in the excretory canals and the alveoli. But Crocq, as early as 1873, tried to prove that every case of parotid inflammation with a general cause is preceded by some inflammation in the mouth, which extends into the gland through the duct of Steno. The recent histologic and bacteriologic researches of Claisse and Dupre confirm this view. In a large number of cases they were able to trace directly the upward extension of the infection from the mouth up into the gland. It is in this way we know that infection finds its way into the liver, the kidneys, the bronchial tubes and the breasts, through the excretory ducts, and it is probably the same with the parotid gland. But this opinion must not be regarded as absolutely settled, although it has been verified in a large number of cases. Duplay, in his Treatise on Pathology, suggested that the infection might arrive by way of the lympathic glands, and in that case it is not the parenchyma of the gland, but the lymphatic ganglia, that are affected. This location of the inflammation must be borne in mind as much as possible when diagnosing; but if it is of lymphatic origin an abcess will form, the opening of which will show an accumulation of pus. But if the inflammation is in the glandular pockets, and is being infected through the excretory duct, the lancet only brings out a few tiny drops of pus, contained in the pockets of the gland into which it penetrates, while the edema, the swelling and the sensation of fluctuation would have made you confident that there was a considerable accumulation of pus there.

It seems as if lancing it had done no good, but in reality it is followed by a fall of temperature and local improvement, and the issue of pus later through the duct of Steno explains the above phenomena and proves that it is a glandular inflammation with the pus secreted in the acini or excretory canals.

My first case was a woman recovering from typhoid fever, who became very much swollen in the parotid region, with edema, and fluctuation accompanied by fever. Convinced that there was a large collection of pus, I opened straight into the parotid gland, and found to my surprise that scarcely anything issued from the incision, a few tiny drops of pus being distinguished with difficulty in the blood that followed. The temperature decreased, however, and a couple of days later I saw pus exuding from the duct of Steno. The swelling diminished, and a permanent cure ensued. Another case was a man of 45, large, alcoholic, suffering from multiple articular rheumatism and acne. The parotid region began to swell, there was edema and fluctuation, and I opened the abcess as before, using a grooved director and passing through the whole gland, without finding any accumulation of pus. But the temperature fell afterward, and a couple of days later pressure on the swelling discharged pus through the duct of Steno into the mouth.

A patient came to me suffering from a urinal abcess, which I lanced. Convalescing from this, he was attacked with erysipelas of the face, for the second time in his life. One injection of Marmorek's anti-streptococcus serum was made and the erysipelas passed away, as it might have done without the injection, perhaps.

An abcess next formed at the spot where the serum had been injected, which I opened and found full of streptococci. A few days later the parotid region began to swell, puffiness and fluctuation ensued, and I opened the swelling, out of which poured a large quantity of pus, also full of streptococci. Evidently in this case I had lanced a ganglion abcess, infected through the lymphatic gland.

These three observations show that infection may reach the parotid glands through the excretory canals or the lymphatic glands, producing in one case what Chassaignac calls a canalicular parotid inflammation, or acinous adenitis, and in the other a simple adenitis. In the latter case a certain amount of pus will follow the knife, but in the former case you will not have the satisfaction of seeing the pus escape. But you need not fear that you have made a mistake in your diagnosis. The pus is scattered through the gland, contained in the acini and excretory canals. Lancing always brings relief, lowers the temperature and promotes recovery.

